# Headspace Volatile Evaluation of Carrot Samples—Comparison of GC/MS and AuNPs-hpDNA-Based E-Nose

**DOI:** 10.3390/foods8080293

**Published:** 2019-07-27

**Authors:** Sara Gaggiotti, Marcello Mascini, Paola Pittia, Flavio Della Pelle, Dario Compagnone

**Affiliations:** Faculty of Bioscience and Technology for Food, Agriculture, and Environment, University of Teramo, via Renato Balzarini 1, 64100 Teramo, Italy

**Keywords:** electronic nose, gold nanoparticles-hairpin-DNA (AuNPs-hpDNA), volatile compounds analysis, sensor array, quartz crystal microbalances (QCMs), carrots

## Abstract

The performances of a quartz crystal microbalances (QCMs) based on an electronic nose (E-nose), modified with hairpin-DNA (hpDNA) for carrot aroma profiling has been evaluated. Solid phase micro-extraction (SPME) headspace sampling, combined with gas chromatography (GC), was used as a reference method. The changes in carrot aroma profiles stored at different temperatures (−18 °C, 4 °C, 25 °C, and 40 °C) were monitored during time up to 26 days. The principal component analysis of the data evidenced the different aroma patterns related to the presence of different key compounds. The output data achieved with the hpDNA-based E-nose were able to detect aroma patterns similar to gas chromatography with mass spectrometry (GC-MS). This work demonstrates that hpDNA has different sizes of loops that can be used for the development of sensor arrays able to detect aroma patterns in food and their changes with advantages in terms of easiness of usage, rapidity, and cost of analysis versus classical methods.

## 1. Introduction

Carrot (*Daucus carota* L.) is a widely used and popular vegetable characterized by main nutritional and health properties and peculiar sensory properties, such as color, taste, and flavor. Carrot consumption has increased over the past years due to the growing importance of their healthy properties related to the presence of a series of bioactive compounds and due to the availability of processed carrot products; among others, the frozen and minimally-processed (chilled, ready-to-eat, ready-to-cook) should be mentioned. 

The volatile profiles of carrots has been extensively studied because they significantly contribute to the characteristic flavor as a result of a complex combination of non-volatile and volatile compounds of various nature and functionality, some of which are present at very low concentration. In particular, the pleasant flavor that contributes to consumers acceptance is due to small saccharide and volatiles, mainly terpenes and sesquiterpenes and also, at much lower concentration, to methyl-pyrazines [1,2]. 

Terpenes constitute the largest class of plant secondary metabolites and are important in determining the quality and nutraceutical properties of horticultural food products, including the taste and aroma of fruits and vegetables. Carrots produce many different mono- and sesquiterpene volatiles in leaf and root tissues and their concentration can represent up to 98% of the total volatile mass [1,3,4]. It is vital to investigate freshness, shelf life, and usability of these products because many terpenes emanate from herbs used in a fresh and dried form as spices and flavoring agents in food processing. Human thresholds for odor, nasal pungency, nasal localization, and eye irritation to some of the selected terpenes are low and, thus, highly sensitive and selective methods for quantification of these compounds is needed [5]. 

One approach for determining the maturity and shelf-life of vegetables consists of sensing the aromatic volatiles released by using electronic olfactory systems. Changes in the aroma profile of carrots are due to metabolic post-harvest reactions, microbial growth and fermentations, enzymatic activity, and chemical reactions, including phenolic and lipid oxidation [6]. 

Instrumental analytical methods, such as gas chromatography (GC), gas chromatography with mass spectrometry (GC-MS), sniffing GC-MS, and/or high-performance liquid chromatography (HPLC), combined with appropriate sample preparation techniques, are routinely employed for quality evaluation in laboratories. The latter analytical techniques, however, can be time-consuming and expensive and require skilled personnel to operate the equipment and interpret the analytical outputs. For online applications or fast screening purposes, it has become increasingly important to develop rapid, robust, reliable, and easy-to-operate alternative analytical techniques for aroma fingerprinting [7]. In the last decade, electronic nose technology has opened the possibility to exploit, from a practical point of view, the information provided by the detection of presence and concentration of components in the headspace in many different application fields. Sensor arrays can be designed considering two aspects: Firstly, sensing materials need to be low cost, facile to synthesize, stable, and highly sensitive, as well as selective [8,9]. Secondly, transducer elements should be sensitive to minute fluctuations of analyte concentrations and should be portable and rapid in response [10,11]. One of the simplest configurations in gas sensing is represented by quartz crystal microbalances (QCMs). The sensing surface of QCMs can be easily modified with organic compounds, taking advantage of the measurement run at room temperature. In previous works, these QCMs modified with organic compounds (peptides) were proven to give satisfactory results in the assay of a large range of foods, such as olive oil [12], chocolate, and candies [13,14], or fruits juices [15]. 

In this study, we demonstrate for the first time that different hairpin-DNA (hpDNA) sequences can be used to develop sensor arrays able to discriminate aroma patterns in food products. HpDNA is a sequence of DNA that has a secondary structure constituted by a stem and loop and has been widely used in liquid sensing. The feasibility of the use of molecularly modelled hpDNA sequences for the realization of sensor arrays based on QCMs has already been reported in a previous work [16], where conditions of the sensors were optimized, and the array was tested on pure compounds. In this study, the evolution of the volatile profile of carrot samples using GC-MS/solid phase micro-extraction (SPME) and an hpDNA based electronic nose (E-Nose) (sensor array) was monitored. The samples were analyzed at different times during storage at different temperatures (−18 °C, 4 °C, 25 °C, and 40 °C). The results obtained demonstrated that the carrot samples showed clear changes in aroma compounds that were identified and monitored using GC-MS and the E-nose with similar performances. Considering the low cost of the QCMs modification, the ease of the procedure, and the rapidity of the assay, this hpDNA based E-nose represents an interesting tool for sense aromas and monitoring food quality.

## 2. Materials and Methods 

### 2.1. Samples Preparation and Chemical Standards

One single batch of fresh carrots (*Daucus carota* L. var. Nantesa) bought from a local farmer located in the Abruzzo region (Italy) was used. Carrots were properly washed in tap water to remove impurities. Samples were then peeled to remove 1 mm of the external layer and cut into slices (≈24 mm diameter and 4 mm thickness). In order to obtain a complete inactivation of enzymes causing degradative reactions during freezing, storage, and thawing, the packaged carrots were blanched at 90 °C for 10 min in a water bath. Processing conditions and sample size was selected from a previous work [4]. Afterwards, aliquots of 3 g of blanched carrots were placed in 20 ml gas-tight vials and hermetically sealed with a gas-tight septum. A total of 24 vials were prepared for each temperature tested (−18 °C, 4 °C, 25 °C, 40 °C), in order to have three replicates (three different vials) for each day of measurement (1, 4, 8, 12, 19, 26 days). Thus, a total of 140 vials for E-nose and GC-MS have been analyzed. Each vial was used only once for either the GC-MS or E-nose measurement and the relative sample was discarded. All reagents and standards were purchased from Sigma−Aldrich (Milan, Italy).

### 2.2. GC-MS/SPME Procedure

A Clarus SQ8S GC-MS (Perkin Elmer, Boston, MA) was used to analyze the headspace of all samples. GC-MS analysis was carried out using 3 g of carrot samples into a 20 mL vial closed with a screw cap and silicone septum. The analysis was carried out at all different temperatures tested. Sampling of the volatile compounds was performed by solid phase micro-extraction (SPME). The sample was kept for 20 min at 40 °C and then exposed to the fiber (SPME fiber 50/30 µm, DVB/CAR/PDMS (Supelco, Bellefonte, PA) for 20 min at a fixed temperature. This procedure was also used to extract the headspace of the standards (octanal, terpinolene, α-terpineol, o-cymene, D-limonene, β-pinene) using 2 μL of pure standards into 20 ml vials. The fiber was then inserted in the desorption chamber where GC analysis was carried out with the following temperature gradient: The column was kept at 40 °C for 6 min, then the temperature was raised up to 250 °C at 4 °C/min. The column used was a capillary GC column (30 m × 0.25 mm i.d, 0.25 µm film thickness). The identification of compounds was carried out using the NIST library and by comparison with standards. The peak area and position were obtained using the Turbo-Mass 6.1.0 program. To evaluate the effect of the volatile compound of the carrots, a relative aroma release/retention index (AR %, Aroma retention/release) was calculated.

### 2.3. Electronic Nose Analysis

The samples and standards analyses were carried out using an Electronic nose UTV (Tor Vergata Sensors Group, Rome, Italy), equipped with a twelve Quartz Crystal Microbalance (QCM) sensor array. QCM sensor modification was carried out using gold nanoparticles (AuNPs) functionalized with hpDNA. A total of 8 hpDNA sequences with different unpaired loops were used to realize the sensor array, as listed in Table S1. An unpaired tetramer loop was purchased from Thermo Fischer Scientifics (Italy); the others were from Integrated DNA technologies (USA). Standard desalted purified oligonucleotides were bought with a thiol spacer with six carbons. AuNPs were synthesized using the trisodium citrate reduction method [17]. Immobilization of the oligonucleotides on the AuNPs surface was carried out covalently using a C6 thiol modifier group attached to 5’ phosphate end of the hpDNA. The QCM sensors modification was achieved by drop casting 5 μL of the hpDNA-AuNPs suspension on each side of the crystal and letting it dry for few minutes. Before the first use, the QCM sensors were completely dried under N_2_ at a flow rate of 2 L/h and stored at room temperature in the dark when not in use. Analysis of aldehydes and terpenoids was carried out using 100 µL of pure standard using a glass lab bottle (100 mL). There were three steps of measurements: 2 min to completely remove the air in the bottle (pre-opening); 10 min to equilibrate the headspace at 25 °C in stationary conditions (formation of headspace), and finally the valves were opened to carry the head-space to the measuring chamber. A steady state was reached between 5–6 min after the start of the measurement. After each measurement, a complete recovery of the initial signal was achieved for all compounds in about 400 s. Analysis of carrot samples was carried out using 3 g of carrots in glass lab bottles (100 mL). Measurements were made on sample storage at different temperatures (−18 °C, 4 °C, 25 °C, and 40 °C) and analyzed on different days (from 1st day to 26th day). Carrots were kept for 5 min before starting the measurement to enrich the headspace of volatile compounds. The headspace was then assayed by the E-nose for 8 min. The frequency shift (ΔF) was taken as an analytical signal. The ∆F represents the difference between the frequency value at the beginning of the measurement and the lowest value reached by the sensor during the measurement. Finally, to highlight the affinity between volatile compounds and sensors, the data were normalized, as reported by Di Natale et al. [18]. 

### 2.4. Statistical Analysis

Statistical analysis of all carrot samples was carried out using XLSTAT software (Addinsoft, New York, NY, USA). Dataset obtained with E-nose were normalized for the sum of the signals of each sensor and, subsequently, for the sum of the signal of the sample as detected by all sensors before the statistical analysis. Principal component analysis (PCA) was used for a multivariate data set from electronic nose sensors array and was also applied on a GC-MS peak area data set. Eventually, a correlation test between molecules identified with GC-MS and the E-nose responses was made. 

## 3. Results and Discussion

### 3.1. Flavor Profile of Carrot Samples Using GC-MS/SPME

In the first step, the GC-MS/SPME method was used to monitor the volatile compound (VOC) changes in carrot samples stored at different conditions. In Table 1, the volatile compounds varying significantly in relative amount (%) during the experimental phase are listed as a function of time and temperature of storage. 

In total, 18 volatile compounds were found and identified by means of the NIST library (over 80% of probability of identity); the majority of carrot volatiles are terpenoids. Considering the literature data on fresh carrots [19], the number of volatile compounds determined in this study on the samples is significantly lower; this can be attributed to the initial blanching step (this pretreatment is commonly used in industrial production to reduce the enzymatic degradation). Shamaila et al. [20] observed a significant change of many volatiles of blanched carrot slices, in particular terpenoids (sabinene, β-pinene, β-myrcene, D-limonene, trans-caryophyllene, α-humulene, β-bisabolene, and α-farnesene), and a decrease by at least 50% within 60 s of blanching.

The mostly represented volatile compounds were α-pinene and γ-terpinolene, which were detected in all samples stored at the different temperatures, except for samples stored at 40 °C on the first day of analysis; butane-2,3-diol, followed by acetoin and lactamide, were formed only for storage at 25 °C from the 8th day and during storage at 40 °C. The presence of acetoin and butane-2,3-diol was attributed to fermentation during the storage, producing degradation of samples. Octanal, determined in refrigerated samples, is a fatty acid-derived volatile and its presence in refrigerated carrot has already been reported by other authors [19].

**Figure 1:**
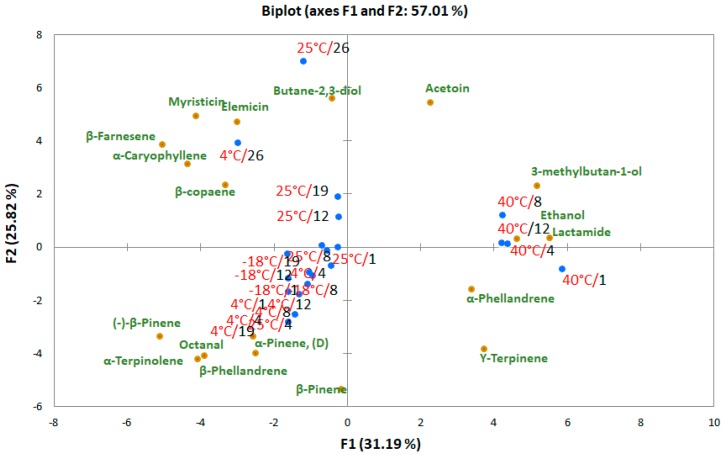
Principal component analysis (PCA) of the gas chromatography with mass spectrometry/solid phase micro-extraction (GC-MS/SPME) analysis of carrot samples stored at different temperatures. Data are expressed in relative abundance (%) before PCA.

Compounds, such as terpenoids and alcohols, were identified by other authors [21] as potential markers of carrot quality. A simple multivariate data treatment was performed using PCA to extract the most important information from the obtained data set. Figure 1 shows a samples (scores) and variables (loadings) plot corresponding to the first two components, which concentrate 57.01% of the total variance (PC1: 31.19% and PC2: 25.82%) of the dataset. 

A bi-plot graph is an interesting tool to represent the changes in the headspace pattern during storage. The analytical data of the headspace composition of carrot samples stored at different temperatures is spatially distinct, and the effect of storage conditions can be clearly observed.

The score plot showed good discrimination among the different storage conditions. In particular, all the samples stored at 40 °C were clearly separated on the first principal component (PC1), while the majority of the rest of the samples were grouped in the 3th quadrant of the bi-plot, including all the samples stored at −18 °C and 4 °C, except the sample assayed on the 26th day (4th quadrant). The carrot samples stored at 25 °C were in the same quadrant from day 1 to day 8 and were present in the 4th quadrant from day 12 to day 26. The discrimination factor of these latter samples can be attributed to higher presence of terpenoids weighing on the second principal component (PC2) (see loadings), while PC1 successfully separated acetoin, lactamide, 3-methylbutan-1-ol, and ethanol. All these compounds, which are critical for a good separation of the carrot samples, since they vary significantly in terms of concentration, are also important in terms of olfactory impact and, thus, sensory attributes. In fact, acetoin, 3-methylbutan-1-ol, and ethanol give a strong odor of fermentation, while terpinolene, β-pinene, (−)-β-pinene, and α-pinene can generate a medium woody, fresh minty, and eucalyptus smell (http://www.thegoodscentscompany.com).

Moreover, the relative amount of the terpenoids varied during storage, probably for the degradation into other components. In fact, it has been previously reported that these compounds are highly thermolabile and sensitive, even at low temperature variation [20,22]. 

The analysis by GC-MS allowed us to evaluate the presence and relative concentration of the most important volatile compounds and to evaluate their change during storage at different conditions (time/temperature). While limited for the identification of only a certain amount of aroma compounds, these results have a key role for the interpretation of the E-nose results.

### 3.2. HpDNA Gas Sensor Array Response to Volatile Compounds (VOCs) 

The hpDNA sequences used in this study were selected using a computational approach developed by our group [16]. The in-silico screening procedure was aimed to test the virtual binding affinities of all possible combinations of tetramer, pentamer, and hexamer single strain DNA (ssDNA) of the hairpin loop vs. four chemical classes (alcohols, aldehydes, esters, ketones). The eight sensors hpDNA array was firstly challenged with pure standards of octanal, hexanal, terpinolene, α-terpineol, o-cymene, D-limonene, and β-pinene to understand the potential discrimination ability of the array. Typical responses for octanal achieved after three months of continuous use of the array are reported in the supplementary material (Figure S1) and confirm the stability and reproducibility of the performed measurements. 

**Figure 2:**
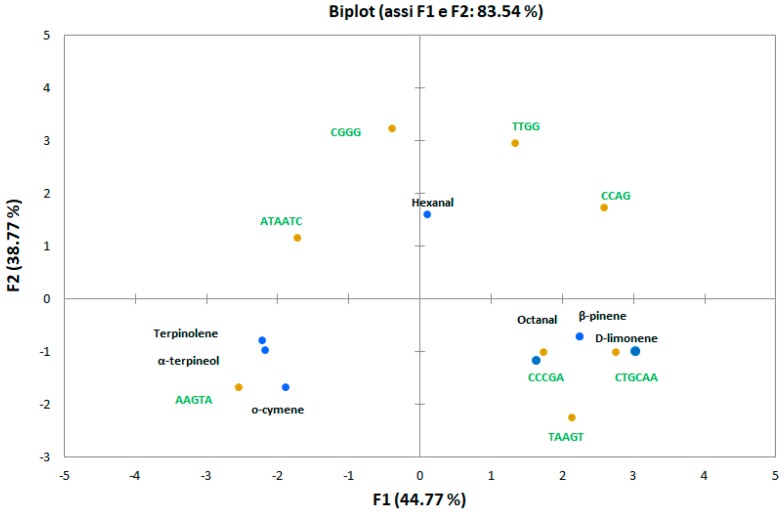
PCA biplot (score and loading) of the normalized quartz crystal microbalance (QCM) response.

The signal obtained (∆F, Hertz) for the different standards was auto scaled and then analyzed using PCA. Figure 2 reports the first and second principal components with 83.54% of variance. The loadings represent, in this case, the contribution of each hpDNA sensor to the principal components and the scores of the VOCs. 

As already reported in a previous work, where other standards were used [16], the hpDNA sequence pentamers and hexamers in the loop have different responses for different VOCs and contribute mostly to the discrimination; sequences with tetramers (CGGG, TTGG, CAGC) seem to be more homogeneous in the response (not spread over the plot). The higher ability to discriminate among the volatile compounds is shown by the first principal component (PC1), which represented 44.77% of the variance. 

Overall, results reported in Figure 2 demonstrated that the hpDNA sensor array system presents some ability to discriminate pure key compounds found in carrot sample headspace in GC-MS analysis, and this allowed its application on testing the responses of the vapor phase of real samples. 

### 3.3. HpDNA Gas Sensor Array Analysis of Carrot Samples

Similar gas sensor arrays, which realized immobilizing peptides onto gold nanoparticles (AuNPs), exhibited a drift of the signal for samples with high water content [12,13,14,15,23]. This effect can be attributed to the AuNPs and can reduce the discriminating ability of the array. The reproducibility and robustness of the measurement using carrots was then tested carefully. 

**Figure 3:**
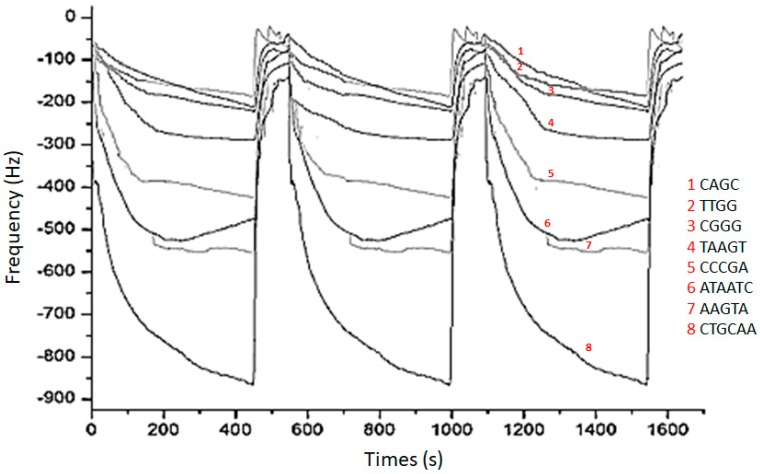
Frequency signal recorded with gold nanoparticles/hairpin-DNA (AuNPs-hpDNA) array (three repetitions performed on the same sample).

In Figure 3, the frequency signal recorded with AuNPs-hpDNA testing carrot samples is reported for a triplicate measurement on the carrot sample. It can be observed that no signal drifts were detected, and the reproducibility was satisfactory; this performance was retained for long time (up to three months) with an intraday RSD in the 15–25% range. 

The absence of any drift can be attributed to the hindered access of the water to the AuNPs due to the densely packed hpDNA.

**Figure 4:**
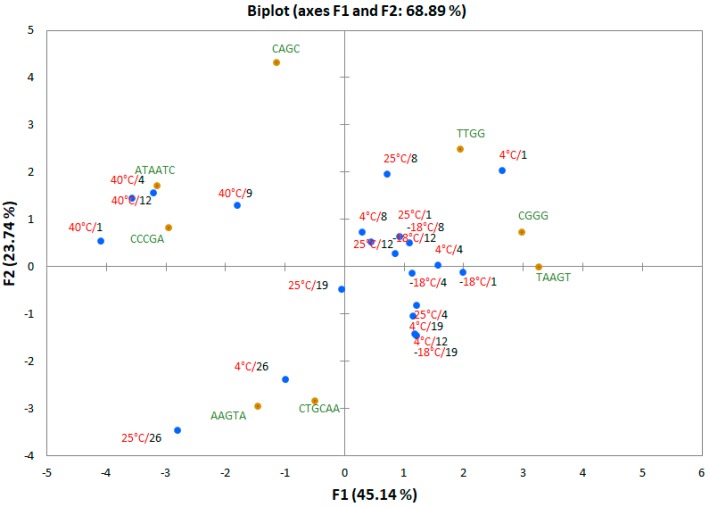
PCA biplot (scores and loadings) obtained with the electronic nose (E-nose) responses of the whole set of carrot samples. Data were normalized before PCA.

The responses of each sensor obtained for the carrot samples were normalized to minimize the effect of different sensitivity and amounts of volatile compounds, then the normalized data were processed by PCA. The obtained PCA, reported in Figure 4, gave 68.89% of the total variance explained by the first two PCs. Carrots samples were well discriminated for GC-MS analysis. 

All the samples stored at 40 °C were in the 4th quadrant. The majority of the other samples were found in the 1st and 2nd quadrants. As observed in the PCA of the results of the GC-MS analysis, samples stored at 4 °C for 26 days and those at 25 °C for 19 and 26 days were separated from the others (3rd quadrant). Only the sample stored at 25 °C for 12 days was not well discriminated in the GC-MS analysis. 

Loadings were well distributed between the quadrants, demonstrating that all the realized hpDNA-based gas sensors contributed to samples discrimination. 

## 4. Conclusions

This study demonstrated, for the first time, the exploitability of an hp-DNA-based gas sensor array to evaluate volatile organic compounds headspace evolution in solid food matrices. The analysis was carried out at different storage temperatures on different days, monitoring possible changes in the aromatic patterns. AuNPs-hpDNA-based gas sensors allowed us to obtain the same discrimination of GC-MS analysis, demonstrating the usability of the employed hpDNA sequences. Indeed, the sensor array proposed results able to track the relevant changes in carrots aroma, confirming that the electronic nose is an instrument that can be used for food quality control with advantages related to a simple preparation of the sample, low cost, and quick analysis.

This work represents the starting point for the use of hpDNA-based gas sensors for food analysis. Indeed, the E-nose proposed represents a new useful analytical tool able to monitor the gases released from vegetables in different storage and ripening stages.

## Figures and Tables

**Table 1:** Results of the gas-chromatography (GC) analysis performed on the carrot samples whole set. Data are expressed as % of the total GC area *.

Volatile Compounds	GC Area (%)																				
	Storage Time (days)																				
	−18 °C					4 °C						25 °C					40 °C				
	1	4	8	12	19	1	4	8	12	19	26	1	4	8	12	19	26	1	4	8	12
α-phellandrene	n.d	n.d	n.d	n.d	n.d	1	1	1	1	1	n.d	n.d **	1	n.d	n.d	1	n.d	7	1	n.d	n.d
β-phellandrene	2	1	2	1	1	3	3	2	1	4	1	n.d	3	1	1	2	n.d	n.d	n.d	n.d	n.d
terpinolene	n.d	n.d	1	1	0	1	1	1	n.d	1	n.d	n.d	1	n.d	n.d	1	n.d	n.d	n.d	n.d	n.d
α-pinene	14	9	12	12	10	14	12	14	14	18	8	12	7	12	11	15	2	n.d	10	15	5
(-) -β-pinene	3	2	3	3	3	3	3	3	3	3	2	2	3	2	2	3	1	n.d	n.d	n.d	2
β-pinene	3	2	3	2	6	5	6	3	3	5	1	2	6	1	1	4	n.d	5	3	3	2
Octanal	1	1	n.d	n.d	n.d	1	1	1	1	n.d	n.d	1	n.d	n.d	n.d	n.d	n.d	n.d	n.d	n.d	n.d
γ-terpinene	7	8	9	10	7	8	9	7	8	4	1	12	7	6	5	7	n.d	21	9	9	5
β-farnesene	1	1	1	1	1	1	1	1	1	n.d	1	1	1	1	1	1	2	n.d	n.d	n.d	n.d
α-caryophyllene	1	1	1	1	2	2	1	1	1	1	5	1	2	1	2	1	2	n.d	n.d	n.d	n.d
β-copaene	2	2	2	n.d	4	2	2	3	n.d	n.d	n.d	2	1	2	2	2	5	n.d	n.d	n.d	n.d
myristicin	1	1	1	1	2	1	1	1	1	1	5	2	1	2	n.d	1	4	n.d	n.d	n.d	n.d
elemicin	n.d	1	n.d	n.d	n.d	n.d	n.d	1	n.d	n.d	4	1	n.d	1	1	n.d	2	n.d	n.d	n.d	n.d
butane-2,3-diol	n.d	n.d	n.d	n.d	n.d	n.d	n.d	n.d	n.d	n.d	n.d	n.d	n.d	2	2	5	13	n.d	n.d	n.d	3
acetoin	n.d	n.d	n.d	n.d	n.d	n.d	n.d	n.d	n.d	n.d	n.d	n.d	n.d	9	3	6	15	5	3	5	6
ethanol	n.d	n.d	n.d	n.d	n.d	n.d	n.d	n.d	n.d	n.d	n.d	n.d	n.d	n.d	n.d	n.d	n.d	5	n.d	n.d	6
lactamide	n.d	n.d	n.d	n.d	n.d	n.d	n.d	n.d	n.d	n.d	n.d	n.d	n.d	n.d	n.d	n.d	n.d	4	11	16	7
3-methylbutan-1-ol	n.d	n.d	n.d	n.d	n.d	n.d	n.d	n.d	n.d	n.d	n.d	n.d	n.d	n.d	n.d	1	2	1	3	3	3

*, (mean value of *n* = 3 repetitions); **, Not detected (n.d).

## Supplementary Materials

The following are available online at https://www.mdpi.com/2304-8158/8/8/293/s1. Figure S1: hpDNA-modified sensors array response vs. octanal after three months from start analysis. (A) Represented the measurements carried out at 25 °C from the 1st day to the 8th day; (B) measurements carried out at 4 °C from the 1st day to the 8th day. Table S1: Sequences of different loop size hpDNA.
