# Supplementary material for: Headspace Volatile Evaluation of Carrot Samples—Comparison of GC/MS and AuNPs-hpDNA-Based E-Nose
Source: Foods. 2019 Jul 27;8(8):293. doi: 10.3390/foods8080293 (PMC6722575; doi:10.3390/foods8080293)
Supplement: Supplementary file 1 [file foods-08-00293-s001.pdf]

Supplementary Materials:

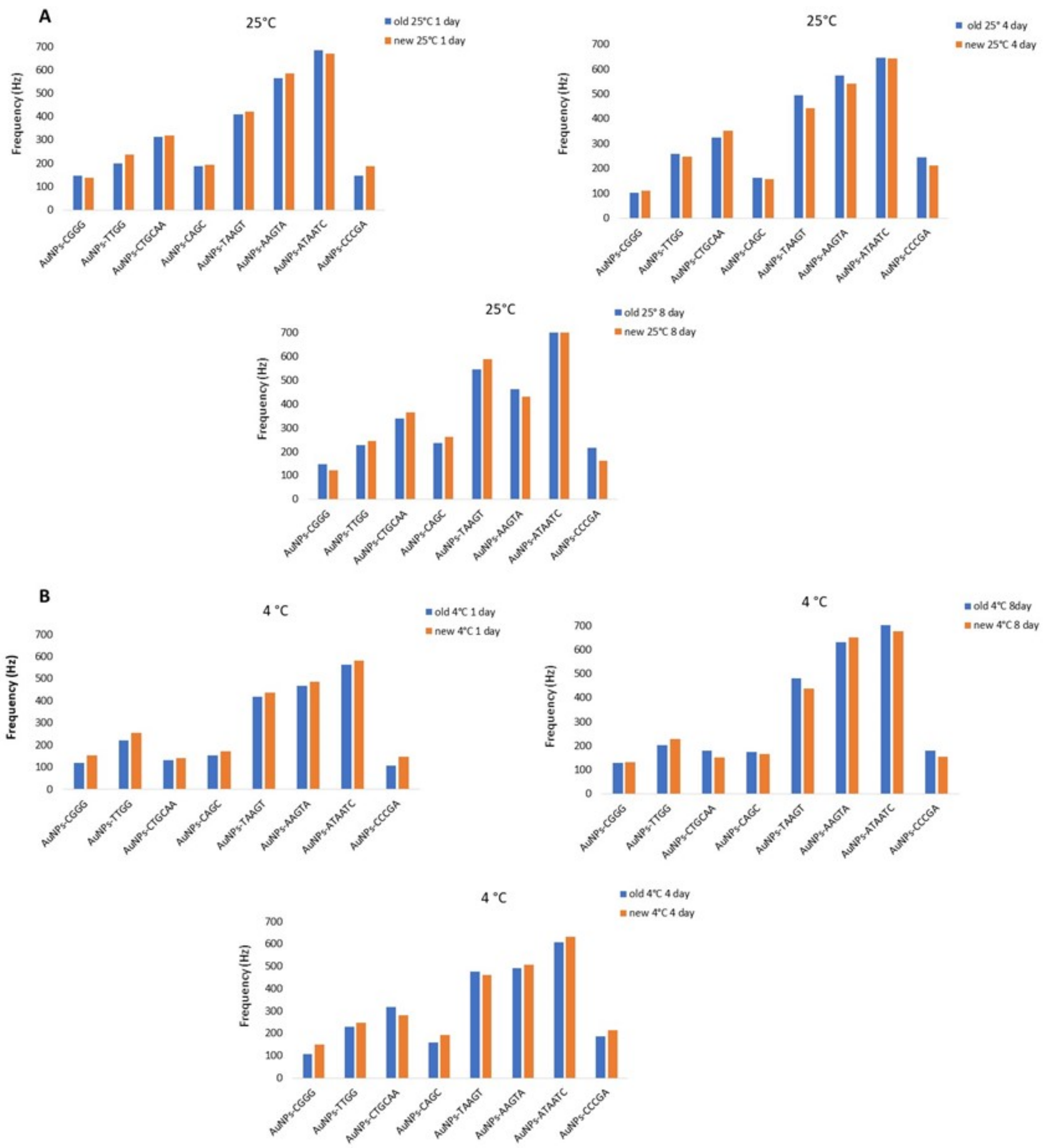

Figure S1. hpDNA-modified sensors array response vs. Octanal after three months from start analysis. (**A**) Represented the measurements carried out at 25 °C from 1th day to 8th day; (**B**) measurements carried out at 4 °C from 1st day to 8th day.

Table S1. Sequences of different loop size hpDNA.

hpDNA	*Sequences*
Loop 4	GAAG-TTGG-CTTC
Loop 4	GAAG-CGGG-CTTC
Loop 4	GAAG-CCAG-CTTC
Loop 5	GAAG-TAAGT-CTTC
Loop 5	GAAG-AAGTA-CCTT
Loop 5	GAAG-CCCGA-CTTC
Loop 6	GAAG-ATAATC-CTTC
Loop 6	GAAG-CTGCAA-CTTC
